# MMP12 Inhibits Corneal Neovascularization and Inflammation through Regulation of CCL2

**DOI:** 10.1038/s41598-019-47831-z

**Published:** 2019-08-09

**Authors:** Marie Wolf, Selene M. Clay, Siyu Zheng, Peipei Pan, Matilda F. Chan

**Affiliations:** 1 Department of Ophthalmology, University of California, San Francisco, California USA; 2 Francis I. Proctor Foundation, University of California, San Francisco, California USA

**Keywords:** Cell migration, Cellular imaging, Mechanisms of disease, Corneal diseases

## Abstract

Following corneal injury, coordinated cellular and protein interactions occur at the wound site to restore tissue homeostasis. Regulation of this response is required to prevent the development of chronic inflammation, abnormal neovascularization, and fibrosis. The chemokine CCL2 and its primary receptor CCR2 are key regulators of the inflammatory and neovascular responses to injury. In this study, we investigated the role of macrophage-associated matrix metalloproteinase 12 (MMP12) in the regulation of CCL2 and CCR2 after corneal wounding. Using two corneal injury models, we examined the temporal and spatial expression of CCL2 and CCR2 in *Mmp12*^−/−^ and wild-type (WT) mice. Our data showed that MMP12 downregulated CCL2 and CCR2 expression in a manner dependent on the timing and mechanism of injury. We also examined the effect of CCL2 on the injury response in *Mmp12*^−/−^ and WT corneas. We found that macrophage infiltration and neovascularization following CCL2 blockade was significantly reduced in *Mmp12*^−/−^ corneas as compared with WT corneas. These findings indicate that MMP12 inhibits corneal inflammation and neovascularization after injury through its regulation of CCL2.

## Introduction

Tissue repair after injury is complex and involves dynamic interactions between inflammatory cells, chemokines, growth factors, matrix metalloproteinases (MMPs), and the extracellular matrix (ECM). Regulation of cellular events is required to restore tissue homeostasis and minimize tissue scarring and pathology^1^.

Neutrophils and macrophages are both recruited at the injury site where they express and release a variety of proteinases into the wound extracellular space^2^. MMP12 (macrophage metalloelastase) is a proteinase secreted from macrophages that was first identified through its elastase activity^3^. The list of MMP12 substrates has since expanded to include other ECM components, as well as non-ECM components, and consequently its role in various cellular processes has likewise grown^4–7^.

We previously found that MMP12 is expressed following corneal injury and protects against the development of corneal fibrosis^8,9^. Chemically injured corneas of *Mmp12*^−/−^ mice, as compared with injured wild-type (WT) corneas, showed increased expression of fibrosis markers, elevated levels of macrophage infiltration, and increased neovascularization^8^. Wounded *Mmp12*^−/−^ corneas also had elevated expression levels of the chemokine CCL2. Prior studies have shown that CCL2 can induce macrophage recruitment to the corneal stroma^8,10,11^ and promote angiogenesis through VEGFA expression^12^. Despite its role in these repair pathways, further characterization of CCL2 activity following corneal injury is lacking.

To analyze the role of CCL2 and its primary receptor CCR2 in corneal repair, we examined the expression of CCL2 and CCR2 in wounded corneas of WT and *Mmp12*^−/−^ mice using two corneal injury models: epithelial injury which affects the superficial epithelial layer, and chemical injury which involves both the epithelial and stromal layers. Because MMP12 *in vitro* has been shown to cleave and inactivate CCL2^5^, we tested the ability of MMP12 to directly inhibit CCL2 *in vivo* by treating wounded WT and *Mmp12*^−/−^ corneas with a neutralizing antibody to CCL2 and examining markers of corneal inflammation and neovascularization.

## Results

### Induced expression of CCL2 and CCR2 following chemical corneal injury is inhibited by MMP12

CCL2 has been shown *in vitro* to serve as substrate for MMP12^5^. We observed *in vivo* CCL2 to play a key role in promoting macrophage recruitment to the corneal stroma following chemical injury^8^. Furthermore, we observed an important role for MMP12 in regulating CCL2 expression^8^. To further characterize MMP12 in the corneal response to injury, we measured the effect of MMP12 on the temporal expression of CCL2 and its receptor CCR2 following chemical injury.

**Figure 1:**
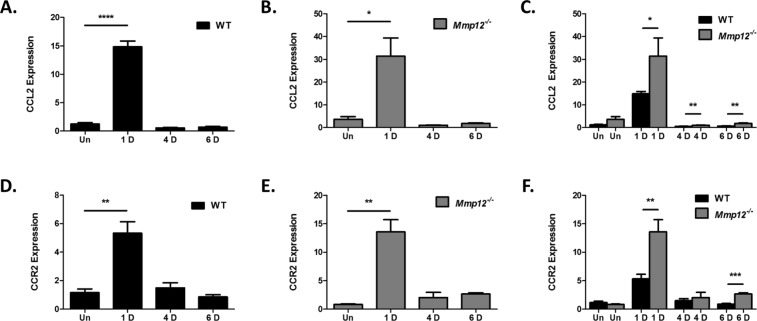
MMP12 inhibits expression of CCL2 and CCR2 following chemical corneal injury. (**A**–**C**) Relative expression levels of CCL2 mRNA in unwounded corneas and chemically wounded corneas of WT and *Mmp12*^−/−^ mice, as determined by qRT-PCR. Expression levels are relative to uninjured WT corneas. Results are relative expression levels (means ± s.e.m.) at time points unwounded (Un; WT N = 12; KO N = 10), 1 day (1D; WT N = 12, KO N = 12), 4 days (4D; WT N = 4; KO N = 4), and 6 days (6D; WT N = 12; KO N = 12) after injury. (**D**–**F**) Relative expression levels of CCR2 mRNA in unwounded corneas and chemically wounded corneas of WT and *Mmp12*^−/−^ mice, as determined by qRT-PCR. Expression levels are relative to uninjured WT corneas. Results are relative expression levels (means ± s.e.m.) at time points unwounded (Un; WT N = 6; KO N = 6), 1 day (1D; WT N = 6, KO N = 6), 4 days (4D; WT N = 4; KO N = 4), and 6 days (6D; WT N = 6; KO N = 6) after injury. ****P < 0.0001, **P < 0.05 and *P < 0.05.

The time course of CCL2 expression in injured corneas of WT mice was examined using a well-established alkali injury model^8,13,14^. We collected uninjured and injured corneas at 1, 4, and 6 days after alkali injury and measured CCL2 mRNA expression using quantitative real-time PCR (qPCR) analysis (Fig. 1A). CCL2 expression was significantly elevated 1 day after injury (15-fold, SD 3.5, *p* < 0.0001) and returned to baseline levels at 4 and 6 days after injury (0.55-fold, SD 0.2 and 0.7-fold, SD 0.3 respectively).

Because we previously found that MMP12 decreases RNA and protein expression of CCL2 following injury, we additionally measured the time course of CCL2 expression in alkali-injured corneas of *Mmp12*^−/−^ mice at 1, 4, and 6 days after injury (Fig. 1B). Similar to WT mice, CCL2 expression was significantly elevated 1 day after alkali injury (31-fold, SD 28, *p* = 0.0034) in *Mmp12*^−/−^ mice and returned to baseline levels at 4 and 6 days after injury (1-fold, SD 0.18 and 1.9-fold, SD 0.65 respectively).

While CCL2 expression was highly expressed 1 day after alkali injury in both WT and *Mmp12*^−/−^ mice, the levels were significantly higher in the *Mmp12*^−/−^ corneas (Fig. 1C). At 1, 4, and 6 days after injury, CCL2 was more highly expressed in injured corneas of *Mmp12*^−/−^ mice compared with WT mice (1 day 15-fold and 31-fold, *p* = 0.041; 4 day 0.55-fold and 1.0-fold, *p* = 0.0011; 6 day 0.7-fold and 1.9-fold, *p* = 0.009; WT and *Mmp12*^−/−^ respectively).

CCL2 can bind the surface chemokine receptors CCR2 and CCR4, though CCR2 is its primary receptor^15^. CCL2 promotes leukocyte trafficking principally through its interactions with CCR2^16,17^. To measure the expression of CCR2 after injury, CCR2 mRNA expression in alkali-injured corneas was measured in WT and *Mmp12*^−/−^ mice at the time points described above. Similar to our CCL2 expression findings, CCR2 expression was found to be significantly elevated 1 day after alkali injury in corneas of WT and *Mmp12*^−/−^ mice (5.3-fold, SD 1.8, *p* = 0.0061 in WT; 14-fold, SD 5.2, *p* = 0.0017 in *Mmp12*^−/−^ mice) and returned to baseline levels at 4 and 6 days after injury (Fig. 1D,E). At 1 and 6 days after injury, CCR2 was more highly expressed in injured corneas of *Mmp12*^−/−^ mice compared with WT mice (5.3-fold and 14-fold respectively, *p* = 0.0044 at 1 day; 0.8-fold and 2.7-fold respectively, *p* = 0.0008 at 6 days) (Fig. 1F). Collectively, these results demonstrate that CCL2 and CCR2 expression is highly upregulated 1 day after chemical corneal injury and that MMP12 reduces CCL2 and CCR2 expression levels.

### Similar spatial expression patterns of CCL2 and CCR2 in corneas of WT and MMP12 KO mice

**Figure 2:**
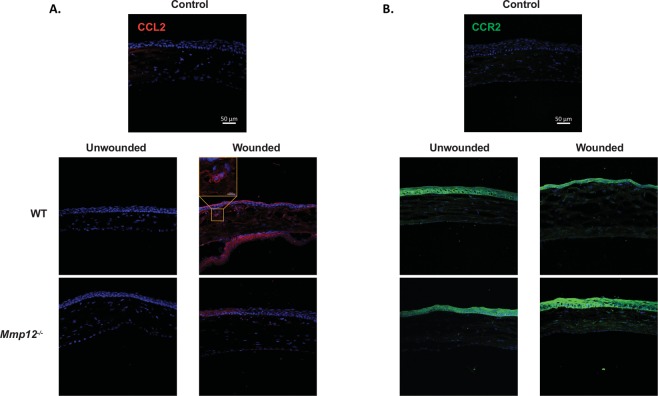
Expression patterns of CCL2 and CCR2 in unwounded and wounded corneas of WT and MMP12 KO mice. (**A**) Immunofluorescence of CCL2 chemokine and (**B**) its receptor CCR2 in unwounded and chemically wounded (2-days after injury) WT and *Mmp12*^−/−^ mouse corneas. Control images represent mouse corneas stained with secondary antibody only and without primary antibody. Nuclei were visualized by staining with DAPI (blue). Scale bars: 50 µm. A magnified image of a wounded WT cornea shows perinuclear expression of CCL2 (orange box). CCL2 staining was visualized in epithelial, stromal, and endothelial layers of wounded WT and *Mmp12*^−/−^ corneas. CCR2 staining was visualized in epithelial and stromal layers of unwounded and wounded WT and *Mmp12*^−/−^ corneas.

In addition to examining the role of MMP12 in the temporal expression of CCL2 and CCR2, we used immunostaining to examine the effect of MMP12 on their spatial expression. CCL2 was not expressed in uninjured WT and *Mmp12*^−/−^ corneas. Following chemical injury, corneas were collected 2 days post-injury when epithelial defects had resolved (Fig. 2A). In both WT and *Mmp12*^−/−^ injured corneas, positive cytoplasmic expression of CCL2 was found in all three layers of the cornea with the highest density in the epithelial layer. Intracellular and cell membrane-associated expression of CCR2 was found in the epithelial and stromal layers of both uninjured and injured WT and *Mmp12*^−/−^ corneas with higher CCR2 expression in wounded corneas (Fig. 2B).

### CCL2 and CCR2 are differentially expressed in epithelial injured corneas of WT and MMP12 KO mice

**Figure 3:**
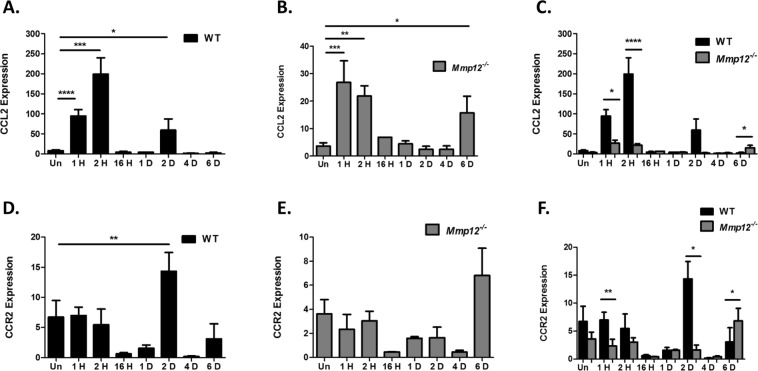
Temporal expression of CCL2 and CCR2 in epithelial injured corneas of WT and MMP12 KO mice. (**A**–**C**) Relative expression levels of CCL2 mRNA in unwounded corneas and epithelial wounded corneas of WT and *Mmp12*^−/−^ mice, as determined by qRT-PCR. Expression levels are relative to uninjured WT corneas. Results are relative expression levels (means ± s.e.m.) at time points unwounded (Un; WT N = 12; KO N = 10), 1 hour (1 H, WT N = 15; KO N = 3), 2 hours (2 H, WT N = 15; KO N = 4), 16 hours (16 H, WT N = 3; KO N = 3), 1 day (1D; WT N = 3, KO N = 3), 2 days (2D; WT N = 14; KO N = 3), 4 days (4D; WT N = 3; KO N = 5), and 6 days (6D; WT N = 5; KO N = 7) after injury. (**D**–**F**) Relative expression levels of CCR2 mRNA in unwounded corneas and epithelial wounded corneas of WT and *Mmp12*^−/−^ mice, as determined by qRT-PCR. Expression levels are relative to uninjured WT corneas. Results are relative expression levels (means ± s.e.m.) at time points unwounded (Un; WT N = 6; KO N = 10), 1 hour (1 H, WT N = 9; KO N = 3), 2 hours (2 H, WT N = 9; KO N = 4), 16 hours (16 H, WT N = 3; KO N = 3), 1 day (1D; WT N = 3, KO N = 3), 2 days (2D; WT N = 8; KO N = 3), 4 days (4D; WT N = 3; KO N = 5), and 6 days (6D; WT N = 5; KO N = 7) after injury. ****P < 0.0001, ***P < 0.005, **P < 0.05 and *P < 0.05.

Corneal alkali injury results in injury to both the epithelial and stromal layers of the cornea. We next sought to determine if MMP12 inhibition of CCL2 and CCR2 expression also occurs in a second injury model involving superficial corneal damage. The corneal epithelial debridement model results in an isolated injury to the surface corneal epithelial layer and leaves the underlying stromal layer intact^14^. Following epithelial debridement of corneas from WT and *Mmp12*^−/−^ mice, we measured CCL2 and CCR2 expression at several time points following injury (1 H, 2 H, 16 H, 1D, 2D, 4D, and 6D), and additionally measured levels in uninjured corneas (Fig. 3). Unlike chemical injuries, isolated corneal debridement injuries resolve after 24 hours, and therefore we included earlier time points (1 H, 2 H, and 16 H) to the time points used in the chemical injury model. In WT mice, CCL2 was highly expressed at 1 H (11-fold, SD 61, *p* < 0.0001), 2 H (24-fold, SD 157, *p* = 0.0002), and 2D (7-fold, SD 106, *p* = 0.046) after epithelial injury (Fig. 3A). In *Mmp12*^−/−^ mice, CCL2 was highly expressed at 1 H (7.5-fold, SD 14, *p* = 0.0001), 2 H (6.1-fold, SD 7.6, *p* = 0.0061), and 6D (4.4-fold, SD 16, *p* = 0.035) after epithelial injury (Fig. 3B). For both WT and *Mmp12*^−/−^ mice, CCL2 expression was bimodal, with elevated levels within the first 2 hours post-injury and at later time points (2D for WT and 6D for *Mmp12*^−/−^ mice) (Fig. 3A,B). Furthermore, CCL2 was more highly expressed early (1 H and 2 H) after injury in WT mice compared with *Mmp12*^−/−^ mice (3.5-fold, *p* < 0.04, and 9-fold, *p* < 0.0001 respectively), but later (6 days post-injury), CCL2 was more highly expressed in *Mmp12*^−/−^ mice compared with WT (5.9-fold, *p* = 0.042) (Fig. 3C).

CCR2 expression following epithelial debridement was highly induced 2 days after injury in WT mice (1.8-fold, *p* = 0.0047) but was at baseline levels at the other time points (Fig. 3D). In *Mmp12*^−/−^ mice, CCR2 expression was elevated at 6 days post-injury but was not statistically significant (Fig. 3E). Similar to induced CCL2 expression following isolated epithelial injury, CCR2 expression was more highly expressed early (1 H and 2D) after injury in WT mice compared with *Mmp12*^−/−^ mice (3.0-fold, SD *p* = 0.034 and 8.75-fold, *p* = 0.044 respectively), but later (6 days post-injury), CCR2 was more highly expressed in *Mmp12*^−/−^ mice compared with WT mice (2.2-fold, *p* = 0.040) (Fig. 3F).

These results show that CCL2 and CCR2 have different expression patterns following the two different injury mechanisms. In both injury models, CCL2 and CCR2 are more highly expressed in *Mmp12*^−/−^ corneas at 6 days post-injury. Interestingly, this time point is similar to the post-injury time point associated with increased macrophage infiltration in *Mmp12*^−/−^ corneas^8^.

### Blocking CCL2 reverses the increased macrophage recruitment in MMP12 KO corneas

**Figure 4:**
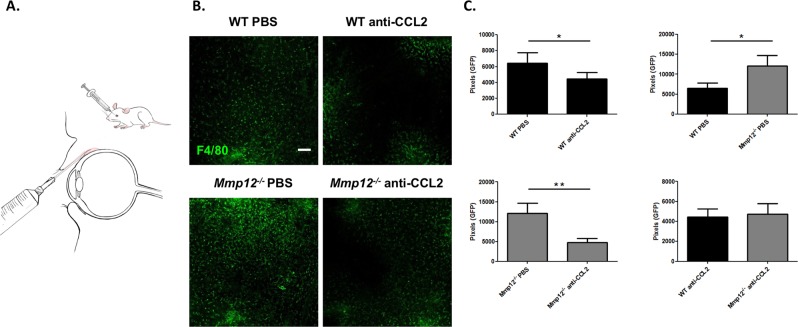
Blocking CCL2 by subconjunctival injection reverses the increased macrophage recruitment in MMP12 KO corneas. (**A**) Primary antibody against CCL2 was delivered adjacent to the cornea into the subconjunctival space. (**B**) Effect of CCL2 neutralization on macrophage infiltration into the corneas of WT and *Mmp12*^−/−^ mice. PBS control or antibody to CCL2 was injected into the subconjunctival space and 2 hours later the corneas were wounded chemically. Corneas were collected 7 days after chemical injury. Representative whole-mount images of F4/80+ levels in central corneas of PBS-treated and anti-CCL2-treated WT and *Mmp12*^−/−^ corneas. Scale bar: 10 µm. (**C**) Quantification of F4/80 levels (pixels) in corneas of PBS-treated and anti-CCL-treated mice. The mean number (±s.e.m.) of F4/80+ cells are shown (WT PBS N = 9; WT anti-CCL2 N = 12; KO PBS N = 9; KO anti-CCL2 N = 15). **P < 0.05 and *P < 0.05.

Macrophages are the major source of MMP12^2^. MMP12 alters the expression of CCL2 and inhibits the accumulation of macrophages in chemically injured mouse corneas^8^. To determine if inhibition by MMP12 of macrophage recruitment to corneal injury is directly mediated through CCL2, we injected a neutralizing antibody to CCL2 or PBS control into the subconjunctival space of injured WT and *Mmp12*^−/−^ mice 2 hours prior to chemical injury (Fig. 4A). At 7 days post-injury, injured corneas of WT mice injected with PBS control had abundant infiltration of F4/80-positive cells into the central cornea (Fig. 4B). Injured corneas of WT mice injected with anti-CCL2 antibody showed a significant 31% reduction in F4/80-positive cells (*p* = 0.012, Fig. 4B,C) and suggests that CCL2 is involved in mediating macrophage recruitment after injury. Injured WT and *Mmp12*^−/−^ mice injected with PBS control showed higher levels of F4/80-positive cells in *Mmp12*^−/−^ corneas compared with WT corneas (1.9-fold higher, *p* = 0.040) (Fig. 4C) and confirmed our prior finding that MMP12 inhibits macrophage recruitment^8^. When *Mmp12*^−/−^ mice underwent subconjunctival injection with either PBS control or neutralizing antibody to CCL2, there was a significant reduction (53.3%, *p* = 0.005) (Fig. 4C) of F4/80-positive cells in the mice treated with anti-CCL2 antibody. This result shows that blocking CCL2 in *Mmp12*^−/−^ mice counteracts the post-injury recruitment of macrophages typically seen in *Mmp12*^−/−^ corneas. Further, injured *Mmp12*^−/−^ corneas treated with anti-CCL2 antibody produced levels of macrophage recruitment similar to those found in injured WT corneas (Fig. 4C). This finding demonstrates that the inhibitory effect of MMP12 on macrophage recruitment to corneal injury is mediated through CCL2.

### Blocking CCL2 reverses the increased neovascularization in MMP12 KO corneas

**Figure 5:**
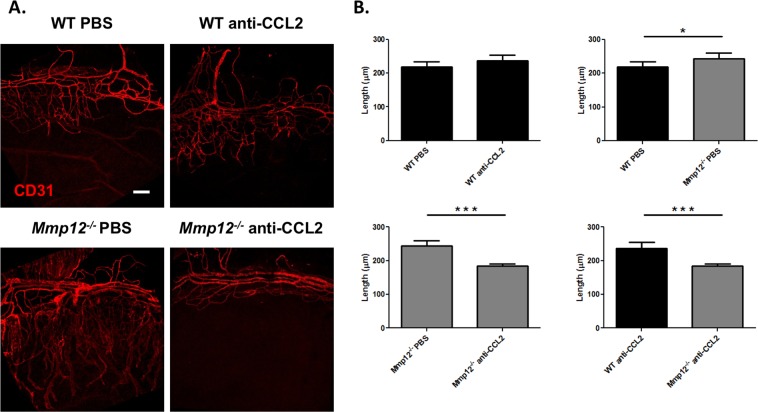
Blocking CCL2 by subconjunctival injection reverses the increased neovascularization in MMP12 KO corneas. (**A**) The effect of CCL2 neutralization on angiogenic endothelium (CD31) in wounded corneas of WT and *Mmp12*^−/−^ mice. PBS control or antibody to CCL2 was injected into the subconjunctival space and 2 hours later the corneas were wounded chemically. Corneas were collected 7 days after chemical injury. Representative whole-mount images of CD31 + limbal vessels in corneas of PBS-treated and anti-CCL2-treated WT and *Mmp12*^−/−^ corneas. Scale bar: 10 µm. (**B**) Quantification of angiogenesis, showing vessel lengths in corneas of PBS-treated and anti-CCL-treated mice. The mean lengths (±s.e.m.) are shown (WT PBS N = 33; WT anti-CCL2 N = 44; KO PBS N = 38; KO anti-CCL2 N = 65). ***P < 0.005 and *P < 0.05.

The observation that blocking CCL2 inhibited macrophage accumulation in the wounded corneas of MMP12 KO mice prompted us to assess whether CCL2 blockade also affected corneal neovascularization. A neutralizing antibody to CCL2 or PBS control was injected into the subconjunctival space in another cohort of chemically injured mice. When injured WT and *Mmp12*^−/−^ mice were injected with PBS control, increased neovascularization was observed in *Mmp12*^−/−^ corneas, indicating that MMP12 can blunt the corneal angiogenic response to injury (218 ± 15 µm; n = 33 versus 244 ± 16 µm; n = 38; *p* = 0.049) (Fig. 5A,B). Injection of PBS control and anti-CCL2 antibody into injured WT mice did not significantly alter corneal neovascular length (218 ± 15 µm; n = 33 versus 237 ± 18 µm; n = 44; *p* = 0.13) (Fig. 5A,B). However, injection of anti-CCL2 antibody into injured *Mmp12*^−/−^ mice resulted in significantly decreased corneal neovascular length compared to WT control mice (244 ± 16 µm; n = 38 versus 184 ± 6 µm; n = 65; *p* = 0.0002). Interestingly, when compared to WT, *Mmp12*^−/−^ mice treated with anti-CCL2 antibody showed significant reduction, and reversal, in corneal neovascularization (237 ± 18 pixels; n = 44 versus 184 ± 6 pixels; n = 65; *p* = 0.0008) (Fig. 5). These findings demonstrate that the inhibitory effect of MMP12 on corneal neovascularization, like its effect on macrophage recruitment is mediated through CCL2.

### Blocking CCL2 partially reverses VEGFA and VEGFB expression in MMP12 KO corneas

**Figure 6:**
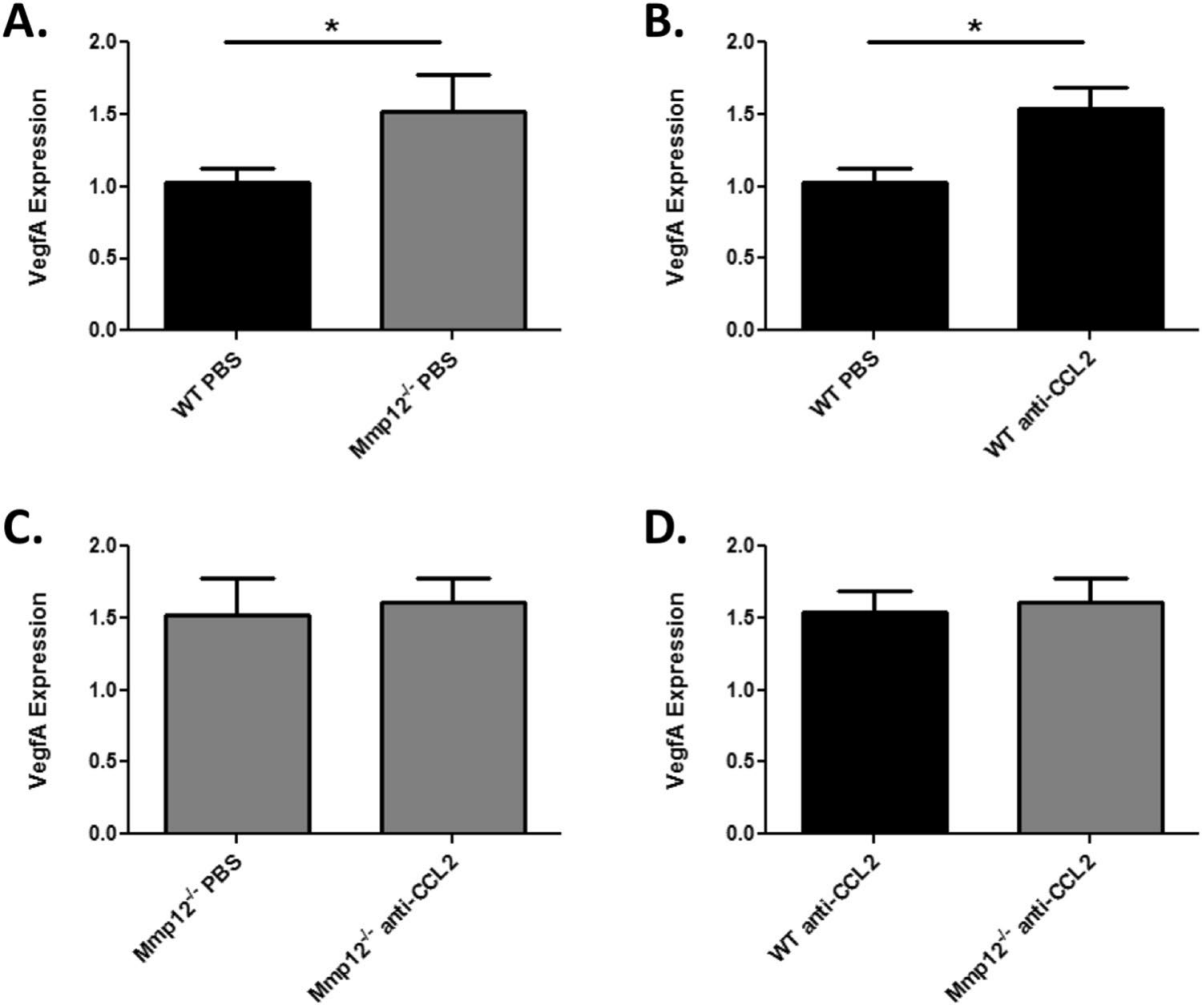
Neutralization with anti-CCL2 antibody partially reverses VegfA expression in MMP12 KO corneas. The effect of CCL2 neutralization on VegfA expression in wounded corneas of WT and *Mmp12*^−/−^ mice. (**A**) VegfA is 1.5 times more highly expressed in wounded corneas of *Mmp12*^−/−^ mice treated with PBS control compared with WT mice (WT PBS N = 8; KO PBS N = 7). (**B**) Treatment of WT mice with anti-CCL2 antibody results in 1.5 times higher VegfA expression (WT PBS N = 8; WT anti-CCL2 N = 9). (**C**) Treatment of *Mmp12*^−/−^ mice with anti-CCL2 antibody results does not affect VegfA expression (1.5 versus 1.6 respectively; KO PBS N = 7 and KO anti-CCL2 N = 9). (**D**) Treatment with anti-CCL2 antibody partially reverses the increased VegfA expression in MMP12 KO corneas compared with WT corneas (1.6 versus 1.5 respectively; KO anti-CCL2 N = 9 and WT anti-CCL2 N = 9). *P < 0.05.

To further define the signaling mechanism underlying the inhibitory effect of MMP12 on CCL2-induced corneal neovascularization, we analyzed expression levels of VegfA. Following alkali injury, corneas of *Mmp12*^−/−^ mice had significantly higher expression of VegfA compared with WT mice (1.5-fold higher, *p* = 0.043) (Fig. 6A). Similarly, injured WT mice treated with anti-CCL2 antibody showed higher VegfA expression than injured WT mice treated with PBS (1.5-fold higher, *p* = 0.005) (Fig. 6B). By contrast, injured corneas of *Mmp12*^−/−^ mice treated with PBS or anti-CCL2 antibody resulted in similar VegfA expression levels (Fig. 6C). Further, anti-CCL2 antibody treatment of injured WT and *Mmp12*^−/−^ mice also resulted in similar VegfA expression levels (Fig. 6D). This result demonstrates that treatment of *Mmp12*^−/−^ mice with anti-CCL2 antibody only partially reverses VegfA expression levels, unlike the full reversal following CCL2 blockade of corneal neovascularization.

**Figure 7:**
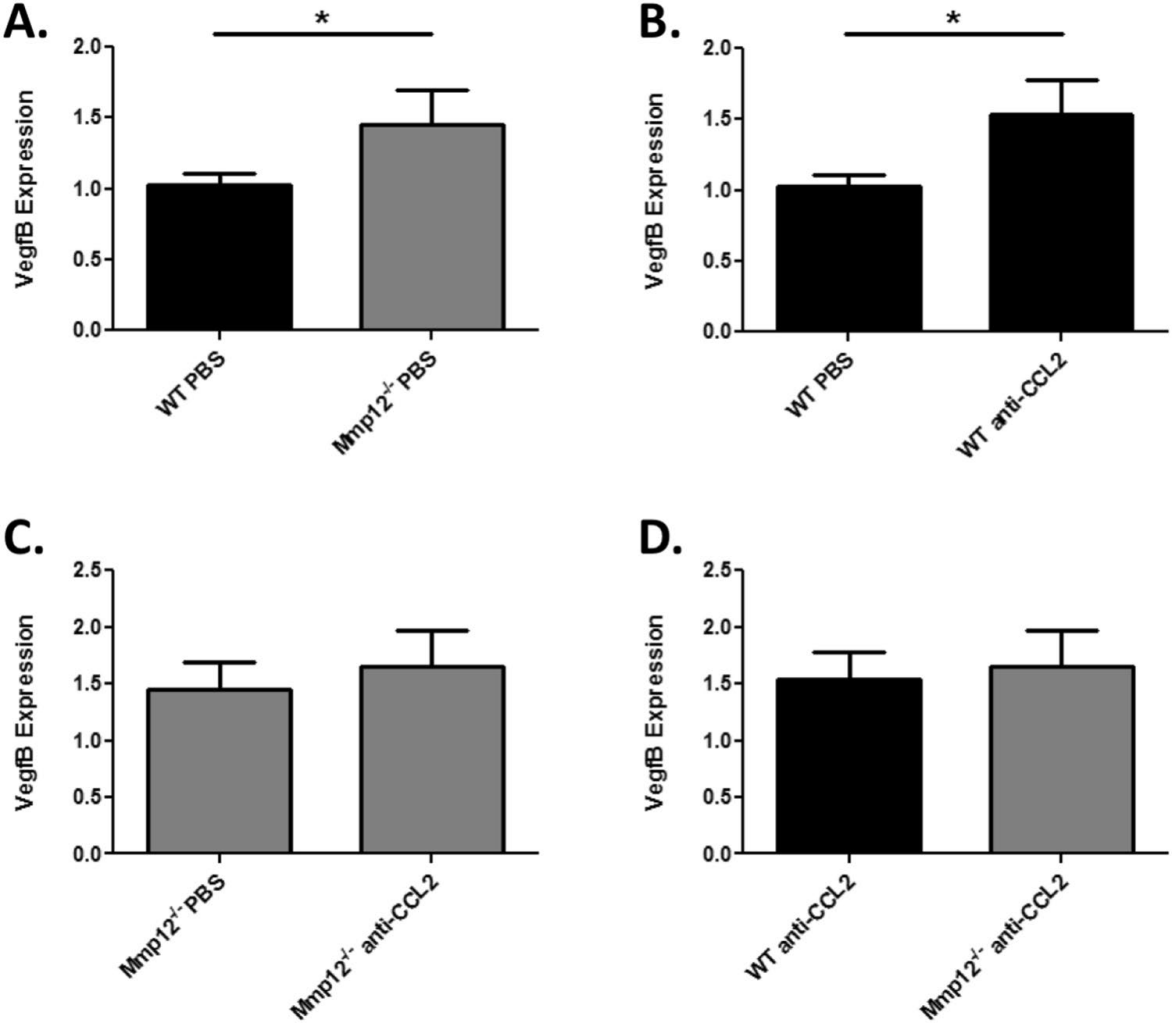
Neutralization with anti-CCL2 antibody partially reverses VegfB expression in MMP12 KO corneas. The effect of CCL2 neutralization on VegfB expression in wounded corneas of WT and *Mmp12*^−/−^ mice. (**A**) VegfB is 1.5 times more highly expressed in wounded corneas of *Mmp12*^−/−^ mice treated with control PBS compared with WT mice (WT PBS N = 8; KO PBS N = 8). (**B**) Treatment of WT mice with anti-CCL2 antibody results in 1.5 times higher VegfB expression (WT PBS N = 8; WT anti-CCL2 N = 9). (**C**) Treatment of *Mmp12*^−/−^ mice with anti-CCL2 antibody does not affect VegfB expression (1.5 versus 1.7 respectively; KO PBS N = 8 and KO anti-CCL2 N = 9). (**D**) Treatment with anti-CCL2 antibody partially reverses the increased VegfB expression in MMP12 KO corneas. (1.7 versus 1.5 respectively; KO anti-CCL2 N = 9 and WT anti-CCL2 N = 9). *P < 0.05.

Because only partial reversal of VegfA expression was found, we decided to also analyze expression levels of VegfB. Interestingly, expression patterns for VegfB were similar to VegfA. PBS-treated corneas of *Mmp12*^−/−^ mice had significantly higher expression of VegfB compared with PBS-treated WT mice (1.5-fold higher, *p* = 0.049) (Fig. 7A). Injured WT mice treated with anti-CCL2 antibody again had a higher expression level of VegfB compared with injured WT mice treated with PBS (1.5-fold higher, *p* = 0.040) (Fig. 7B). *Mmp12*^−/−^ mice treated with anti-CCL2 antibody showed similar VegfB expression levels as *Mmp12*^−/−^ mice treated with PBS (Fig. 7C). Anti-CCL2 antibody treatment of injured WT and *Mmp12*^−/−^ mice also resulted in similar VegfB expression levels (Fig. 7D). These results indicate that the CCL2-mediated effect of MMP12 on corneal neovascularization occurs through the partial reversal of VegfA and VegfB expression.

### MMP12 regulation of CCL2, CCR2 and VEGF protein levels

**Figure 8:**
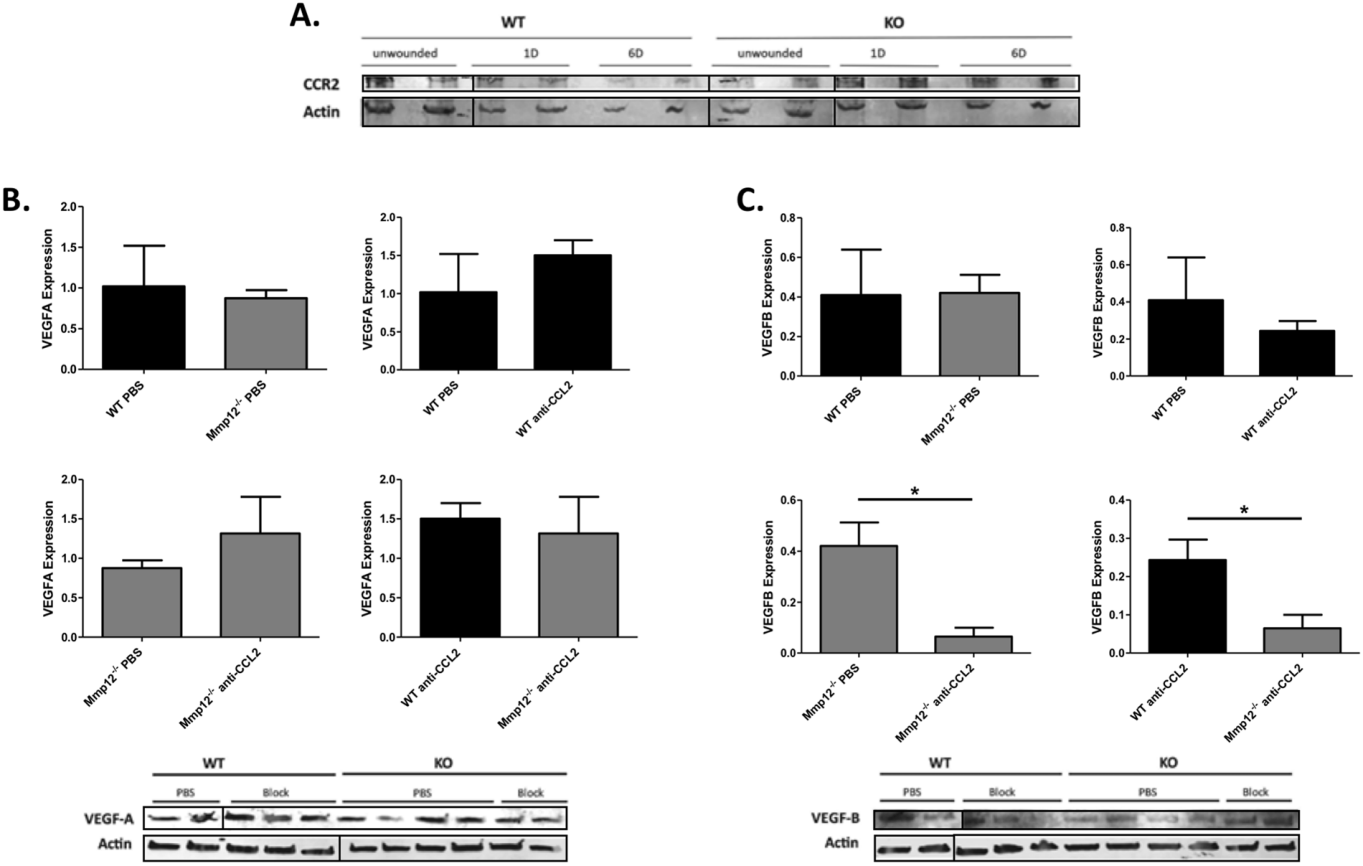
Expression patterns of CCR2, VEGFA, and VEGFB protein in wounded corneas of WT and MMP12 KO mice. (**A**) Protein expression levels of CCR2 and actin in unwounded and wounded corneas of WT (N = 8 per lane) and *Mmp12*^−/−^ (N = 8 per lane) mice 1 and 6 days post-chemical injury, as determined by Western blot analysis. Full-length blots are presented in Supplementary Fig. 1A. (**B**,**C**) The effect of CCL2 neutralization on VEGFA and VEGFB protein expression in WT and *Mmp12*^−/−^ mice at 7 days post-chemical injury. Treatment of WT and *Mmp12*^−/−^ mice with PBS or anti-CCL2 antibody had no significant effect on VEGFA expression. Treatment of *Mmp12*^−/−^ mice with anti-CCL2 significantly decreased VEGFB protein expression compared with PBS-treated *Mmp12*^−/−^ mice (0.42 versus 0.065 respectively). VEGFB expression was decreased more in WT mice compared with *Mmp12*^−/−^ mice following treatment with anti-CCL2 (0.24 versus 0.065 respectively). *P < 0.05. Full-length blots are presented in Supplementary Fig. 1B,C. While we had to use several gels to fit all samples, they all derive from the same experiment and gels/blots were processed in parallel.

The results above show MMP12 regulation of CCL2, CCR2, and Vegf mRNA expression following corneal injury. For CCL2, we found a 2.1-fold increase in CCL2 mRNA levels from corneas of *Mmp12*^−/−^ mice as compared to WT mice at 1 day post-chemical injury (Fig. 1C). This change in mRNA expression is similar to the 1.6-fold increase in CCL2 protein levels from corneas of *Mmp12*^−/−^ mice wounded 1 day prior by chemical injury, compared to WT wounded corneas that we observed in our prior studies^8^. To test whether MMP12 also regulates CCR2 and VEGF protein levels in a manner comparable to mRNA levels, we quantified and compared CCR2 and VEGF protein levels in WT and *Mmp12*^−/−^ corneas following chemical injury (Fig. 8).

Similar to CCR2 mRNA expression, we found low levels of CCR2 protein expression in wounded WT and *Mmp12*^−/−^ corneas 1 and 6 days post-chemical injury (Fig. 8A). Western blot assays also confirmed the expression of both VEGFA and VEGFB protein 7 days post-chemical injury (Fig. 8B,C). Injured WT and *Mmp12*^−/−^ mice injected with PBS control had similar levels of VEGFA and VEGFB protein expression. WT mice injected with a neutralizing antibody against CCL2 showed no change in VEGFA and VEGFB protein expression. *Mmp12*^−/−^ mice injected with a neutralizing antibody against CCL2 had no change in VEGFA expression but had significantly reduced VEGFB expression (6.5-fold lower, *p* = 0.032). Injured WT and *Mmp12*^−/−^ mice injected with a neutralizing antibody against CCL2 also had no change in VEGFA expression but significantly reduced VEGFB expression (3.7-fold lower, *p* = 0.047). This difference in Vegf mRNA and VEGF protein expression patterns at 7 days post-chemical injury may be due to differences in timing of mRNA and protein expression. However, the significant reduction in VEGF protein expression in *Mmp12*^−/−^ mice compared to WT mice following CCL2 neutralization on corresponds to the significant reduction in corneal neovascularization in *Mmp12*^−/−^ mice at 7 days post-chemical injury following CCL2 block (Fig. 5).

## Discussion

Tissue repair after injury is a complex and dynamic process that facilitates regeneration, though when the immune response becomes dysregulated and chronic, fibrosis with tissue dysfunction can result. In the cornea, chronic inflammation and fibrosis manifest as progressive visual impairment and blindness. In order to study how key chemokines are regulated following injury, we have used corneal wounding models. We previously demonstrated higher expression of fibrotic markers and increased levels of angiogenesis in chemically wounded corneas of *Mmp12*^−/−^ mice compared with wounded corneas of WT mice^8^. Wounded corneas of *Mmp12*^−/−^ mice also had greater macrophage infiltration and altered expression of CCL2^8^. Though CCL2 has been shown to have a significant role in mediating macrophage recruitment to the corneal stroma and promoting angiogenesis following injury^10,11,18^, the factors involved in regulating CCL2 have not been established.

**Figure 9:**
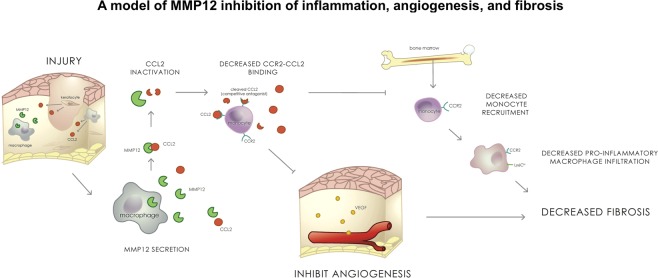
A model of MMP12 regulation of the CCL2-CCR2 signaling axis. Our data suggest that MMP12 and CCL2 are both secreted after epithelial and chemical injuries of the cornea. MMP12 inhibits CCL2 expression and reduces CCL2-CCR2 binding. This leads to reduced monocyte recruitment and reduced angiogenesis which prevents the development of corneal fibrosis.

Results of the present study describe the temporal-spatial expression of CCL2 and its primary receptor, CCR2, following corneal injury and demonstrate regulation of CCL2 by MMP12. We found the temporal expression of CCL2 and CCR2 varies depending on the type of corneal injury. Following chemical injury in which the epithelial and stromal layers of the cornea are injured, CCL2 and CCR2 are both expressed early with high expression levels observed one day after injury in WT mice. Following corneal debridement injury in which only the epithelial layer of the cornea is injured, CCL2 expression was biphasic with elevated levels at early (1 and 2 hours) and later (2 days) time points while CCR2 expression was delayed (2 days post-injury). Using MMP12 null mice, we found that MMP12 regulated CCL2 and CCR2 expression but its regulation depended on the mechanism of injury and timing. CCL2 and CCR2 expression levels were significantly higher in chemically injured *Mmp12*^−/−^ corneas compared with WT corneas at all time points tested. By contrast, CCL2 and CCR2 expression levels were significantly lower in debrided *Mmp12*^−/−^ corneas at early time points but higher levels were found in *Mmp12*^−/−^ corneas 6 days after injury. Furthermore, blocking CCL2 reduced macrophage infiltration and neovascularization to a greater extent in chemically wounded *Mmp12*^−/−^ corneas compared with WT corneas, suggesting that MMP12 directly regulates these processes through CCL2. Collectively, our findings support a mechanism where MMP12 directly inhibits the CCL2 and CCR2 signaling axis and results in reduced macrophage infiltration and angiogenesis (Fig. 9).

CCL2 and CCR2 have critical roles in directing the inflammatory and repair response following corneal injury. CCL2 promotes macrophage recruitment to the corneal stroma following chemical injury^8,10,11^ and viral infection^19–21^, and injured corneas of CCL2-knockout mice exhibit deficient macrophage infiltration^11^. Following corneal epithelial injury, two corneal macrophage populations (CCR2^+^ and CCR2^−^) are observed and have different roles in the corneal repair process^22^. CCR2^+^ corneal macrophages express representative genes of M1 macrophages and promote inflammation during the early stage of wound healing while CCR2^−^ corneal macrophages express representative genes of M2 macrophages and inhibit inflammation during the later stage of repair^22^. Our results show that CCL2 and CCR2 are differentially expressed in *Mmp12*^−/−^ corneas compared with WT corneas following two forms of corneal injury. Furthermore, whether MMP12 inhibits or promotes CCL2 and CCR2 expression depends on both the type and timing of injury. A novel subset of restorative macrophages that promote tissue remodeling has recently been identified in a hepatic fibrosis model^23^. This subset of CD11B^hi^ F4/80^int^ Ly6C^lo^ macrophages falls outside of the M1/M2 classification and highly expresses MMP12^23^. This finding supports further studies to determine whether MMP12 has a functional role in modifying macrophage phenotypes during tissue repair and remodeling.

CCL2 and CCR2 also have important roles in directing the angiogenic response to corneal injury^10,24,25^. The normal cornea is avascular and has angiogenic privilege, but certain forms of corneal injury can induce an angiogenic repair response^26^. In a model of inflammatory angiogenesis in the rat cornea, whole transcriptome gene expression analysis found CCL2 and Cxcl5 to be the most up-regulated factors during active angiogenesis^24^. A model of endogenous resolution of inflammation-induced corneal angiogenesis found down-regulated CCL2 expression during capillary regression^25^. Additionally, CCR2-deficient mice have reduced alkali-induced corneal neovascularization compared with WT mice^10^. CCL2 is recognized as an angiogenic chemokine through its effects on VEGFA expression^12^. VEGFA is a potent stimulator of neovascularization^27,28^, and knock-down of VEGFA expression by small hairpin RNA against VEGFA results in the inhibition and regression of corneal neovascularization^29–31^. We found that the CCL2-mediated effect of MMP12 on corneal neovascularization occurs through the partial reversal of VegfA and VegfB mRNA expression, suggesting the anti-neovascular effects of MMP12 involve non-VEGF dependent mechanisms as well. Topical steroids are the mainstay treatment for corneal neovascularization^32^ and have an expression profile than greatly differs from agents that target VEGF. Relative to anti-VEGF therapy, topical steroid treatment with dexamethasone upregulates C3, Ctgf and C1s expression and downregulates Cxcl5, Reg3g and Ccl2 expression^33,34^. As several complement factors (C3, C3a, C3b, iC3b, C4b, and C5a) are known to be substrates for MMP12^35^, further studies are needed to determine if the non-VEGF anti-angiogenic effects of MMP12 are mediated through these complement pathway factors.

The biological roles of MMPs have traditionally been associated with the degradation of ECM proteins^36^. More recently, degradomics studies and proteomics screens have expanded and diversified the MMP substrate repertoire to include signaling molecules, including 54 chemokines^4^. MMPs can proteolyse and cleave chemokines, altering their bioactive properties and thereby regulating their activity in signaling pathways^4^. MMP12 *in vitro* is able to cleave and inactivate CCL2^5^. Our *in vivo* studies show that MMP12 can inhibit CCL2 function, suggesting the ability of MMP12 to cleave and inactivate CCL2 *in vivo*. As proximity of MMPs to their substrates can vary both temporally and spatially, the probability of substrate cleavage by MMP12 should be determined by tracking the levels of MMP12 and its substrate portfolio over time^37^. CCL2 is produced by many cell types, including fibroblasts, endothelial, epithelial, smooth muscle, mesangial, astrocytic, monocytic, and microglial cells^15^. In the cornea, CCL2 is expressed by epithelial keratocytes^38^ and has been shown to be upregulated after epithelial scrape injury^39^. Our results confirm the upregulated expression of CCL2 following epithelial injury and demonstrate differential expression levels depending on the type of injury as well as time point after injury. MMP12 is also expressed by several cell types including myofibroblasts^40^, hypertrophic osteoclasts^41^, epithelial^42,43^, and vascular smooth muscle cells^41^. In the cornea, MMP12 is expressed by epithelial cells^43^ and keratocytes^44^. While they are expressed by several cells types, macrophages are the major source of both CCL2^15^ and MMP12^3^. Following epithelial injury, our results show that CCL2 and CCR2 are both highly expressed 2 days post-injury, a time point where MMP12 is highly expressed^9^. Following chemical injury, results here show that CCL2 and CCR2 are highly expressed early (1 day) after injury, similar to MMP12 which is also expressed early (2 days) after injury^8^. The similar proximity and temporal expression of MMP12 and its substrate CCL2 after corneal injury supports *in vivo* regulation of CCL2 by MMP12.

In summary, regulation of the CCL2-CCR2 signaling pathway is an important determinate of the corneal fibrotic and angiogenic response to injury. Our two corneal injury models showed inhibition of CCL2 and CCR2 expression by MMP12 and downstream effects on corneal macrophage infiltration and neovascularization. Our findings provide new insights into chemokine regulation following corneal injury and contribute to our understanding of tissue repair and remodeling. This research may reveal new targets for therapeutic intervention.

## Materials and Methods

### Mice

Mice homozygous for the null allele of the MMP12^45^ were genotyped using published protocols and were backcrossed to FVB/n. All experiments were performed with 6–12 week old male and female mice and sibling wild-type littermates served as controls. Mice were maintained under pathogen-free conditions in the University of California San Francisco barrier facility. All animal experiments were conducted in accordance with procedures approved by the University of California San Francisco Institutional Animal Care and Use Committee (Protocol #AN170102).

### Animal models of injury

Corneal alkali burn and epithelial debridement injuries were performed on mice as previously described^8,13^. Mice were anesthetized by isofluorane inhalation (Baxter Pharmaceutical, Deerfield, IL) and by topical application of 0.5% Proparacaine (Akorn Inc., Buffalo Grove, IL) placed on the cornea. Alkaline burn injuries were created by applying filter paper 2.5 mm in diameter soaked in 0.1 N NaOH (Sigma, St. Louis, MO) for 30 seconds to the central cornea followed by rinsing with 250 μl of phosphate buffer saline. After the chemical burn treatment, topical 0.5% Proparacaine was again placed on the cornea for anesthesia. Corneal epithelial debridement injuries were created by using a 1.5 mm trephine (Beaver-Visitec, Waltham, MA) to demarcate the central cornea and the trephine mark was visualized under a stereomicroscope (Leica Biosystems Inc., Wetzlar, Germany). The epithelium within the trephine mark was then removed down to the basement membrane using an Algerbrush II (Katena Products, Inc., Denville, NJ). After the epithelial debridement, topical 0.5% Proparacaine was again placed onto the ocular surface for anesthesia. For both types of injuries, the right eye was wounded and the left eye was left intact as a contralateral control.

### RNA and real-time PCR

Corneal tissue was harvested and stored in 200 uL RNAlater overnight. Total RNA was extracted using homogenization and the Ambion PureLink RNA Mini Kit. For cDNA synthesis, total RNA was reverse transcribed using SuperScript III Reverse Transcriptase (Invitrogen, Carlsbad, CA). For real-time PCR analysis, the expression levels of, CCL2, CCR2, VEGFA, VEGFB, and HPRT were determined using an Applied Biosystems 7500 Instrument (Carlsbad, CA) and cDNA was measured from duplicate samples. Real-time PCR reactions included the following: 20 ng of diluted reverse transcription product, 2X SYBR Green PCR Master Mix (Applied Biosystems, Carlsbad, CA), and 250 nM of each forward and reverse PCR primer. For assays, reactions were incubated at 50 °C for 2 min, 95 °C for 10 min, 40 cycles at 95 °C for 15 s followed by 60 °C for 1 min, and then 95 °C for 15 s. Relative quantification of expression was calculated with the 2−ΔΔCt method and the cycle threshold difference corrected for HPRT. Data are presented as fold change in gene expression relative to uninjured WT corneas and normalized to HPRT. All experiments were performed in duplicate Primer sequences are listed in Table S1 and were purchased from Integrated DNA Technologies (San Diego, CA).

### Whole mount staining and confocal microscopy

Eyes were enucleated and the corneas were dissected to remove the lens, iris, and retina. Four incisions were made equal distances apart to aid in flattening the corneas. Corneal tissue was then fixed with 4% paraformaldehyde in PBS at 4 °C overnight, washed twice in PBS, postfixed with chilled 100% acetone for 20 min at room temperature, washed twice in PBS, and blocked overnight at 4 °C in blocking buffer (PBS + 0.8% Triton X-100 + 2% goat serum). Immunostaining was performed using primary antibodies against F4/80 (MF48000, Invitrogen, Carlsbad, CA), and CD31 (Clone 2H8, Millipore, Hayward, CA) overnight at 4 °C. Tissues were washed in PBS + 0.8% Triton X-100 then incubated with secondary antibodies of the appropriate class at 4 °C overnight (Alexa Fluor 488, Invitrogen, Carlsbad, CA; Dylight 549, Jackson ImmunoResearch Laboratories, West Grove, PA). Routine protocols included corneas stained with an isotype control or secondary antibodies alone. Corneas were then placed epithelial side-up, Fluoro-Gel mounting medium (Electron Microscopy Science, Hatfield, PA) was added, and coverslips were placed.

A confocal laser-scanning microscope (LSM 700; Zeiss) was used to image the localization of Alexa Fluor 488 and Dylight 549 in the central and peripheral cornea respectively. Optical sections (interval of 5um) of confocal epifluorescence images were acquired sequentially with a 10x objective lens with image acquisition software (Zen, Zeiss). Eight optical sections were merged and viewed en face. For F4/80 whole-mount staining images, the number of pixels per color was determined. In addition, for CD31 staining, the lengths of blood neovascularization were calculated using NIH ImageJ software^46^ and the innermost vessel of the limbal arcade was used as the border. For each corneal sample, the lengths of the five longest vessels were measured.

### Immunofluorescence staining and confocal microscopy

For immunofluorescence staining, uninjured and 2 day post-chemical injury globes were collected, immersed in OCT compound, and flash frozen. Ten micron cryosections were prepared and fixed in chilled acetone for 10 minutes. Sections were then air dried for 30 minutes, washed in PBS, and then blocked using 5% goat serum in PBS containing 0.01% Triton X-100 for 1 hour at room temperature. Sections were incubated with primary antibodies (mouse anti-mouse CCL2, NBP2-22115 1:100 dilution; rabbit anti-mouse CCR2, NBP1-48338 1:100 dilution, Novus Biologicals, Littleton, CO) at 4 °C overnight. After 5 washes with PBS, sections were incubated with secondary antibodies (AlexaFluor 546 anti-mouse and AlexaFluor 488 anti-rabbit, Thermo Fisher Scientific, Waltham, MA) for 1 hour at room temperature. After 5 washes with PBS, the sections were stained with DAPI diluted 1:1000 and mounted with Fluoro-Gel (Electron Microscopy Sciences, Hatfield, PA). Images were obtained using confocal laser-scanning (LSM 700; Zeiss).

### *In vivo* inhibition of CCL2

For inhibition of CCL2, a neutralizing polyclonal goat antibody to mouse CCL2 (R&D Systems, Minneapolis, MN) or PBS control was injected into the subconjunctival space of littermate FVB WT and *Mmp12*^−/−^ mice 2 hours prior to corneal chemical injury. Chemical injuries were created by topical application of 0.1N NaOH to corneas as described above. Corneas were collected 7 days after injury and processed for either qPCR analysis or for whole mount immunostaining using primary antibodies against F4/80 and CD31 and secondary antibodies conjugated with Alexa Fluor 488 and Dylight 549. Imaging was performed as described above. NIH ImageJ software was used to quantify the number of pixels of F4/80 stained images and to measure lengths of neovascularization of CD31 stained images.

### Protein assays

Total protein from corneas was extracted on ice with freshly added proteinase inhibitors in RIPA lysis buffer (Thermo Fisher Scientific, Waltham, MA, USA). For VEGF assays, a total of 10 μg/lane protein extract was loaded into a 4–15% SDS-polyacrylamide gel and transferred to PVDF membranes (Bio-Rad Laboratories). Non-specific binding was blocked using 5% BSA in phosphate buffered-saline with Tween-20 (PBS-T) for 1 hour at room temperature. The membrane was incubated with rabbit anti-VEGFA (ab46154; Abcam, Cambridge, MA, USA) or goat anti-VEGFB (AF751; R&D Systems, Minneapolis, MN, USA) primary antibody at 4 °C overnight. IRDye 800CW goat anti-rabbit IgG (Cat. No. 925–32211; LI-COR, Lincoln, NE, USA) and IRDye 800CW donkey anti-goat IgG (Cat. No. 925–32214; LI-COR, Lincoln, NE, USA) respectively were used as the secondary antibody, and rabbit anti-actin antibody (ab179467; Abcam) was used as an internal standard. For CCR2 assays, total protein from 8 pooled corneas per wounding condition was extracted. A total of 77.8 ug/lane protein extract was loaded into a 4–15% SDS-polyacrylamide gel and transferred to PVDF membranes (Bio-Rad Laboratories). The membrane was blocked using 5% BSA in phosphate buffered-saline with Tween-20 for 1 hour at room temperature followed by overnight incubation with rabbit anti-CCR2 (ab203128; Abcam) primary antibody at 4 °C. IRDye 800CW goat anti-rabbit IgG (Cat. No. 925–32211; LI-COR) was used as the secondary antibody, and rabbit anti-actin antibody (ab179467; Abcam) was used as an internal standard. Membranes were imaged using the LiCor Odyssey 9120 Imaging System (LI-CORE Biosciences, United States). Bands were detected using 700 nm and 800 nm channels. Blots were analyzed using ImageJ software and background densities were subtracted from VEGF band densities and normalized to the values for the Actin control.

### Statistical analysis

Statistical analysis was performed with two-tailed t-tests to compare mean values (Prism, GraphPad Software, La Jolla, CA). A p-value less than 0.05 was considered statistically significant.

## Supplementary information

Supplementary Information

## Data Availability

All data generated or analyzed during this study are included in this published article.
