# Supplementary material for: MMP12 Inhibits Corneal Neovascularization and Inflammation through Regulation of CCL2
Source: Sci Rep. 2019 Aug 9;9:11579. doi: 10.1038/s41598-019-47831-z (PMC6689067; doi:10.1038/s41598-019-47831-z)

SUPPLEMENTAL INFORMATION

MMP12 Inhibits Corneal Neovascularization and Inflammation through Regulation of CCL2

Marie Wolf[1], Selene M. Clay[1], Siyu Zheng[1], Peipei Pan[1], and Matilda F. Chan[1,2]

[1]Department of Ophthalmology, University of California, San Francisco, California
[2]Francis I. Proctor Foundation, University of California, San Francisco, California

Running Title: Regulation of corneal neovascularization and inflammation by CCL2

*Address correspondence to:
Matilda F. Chan, M.D., Ph.D. (designee for communication)
UCSF Department of Ophthalmology
Francis I. Proctor Foundation
Surgical Research Laboratory
Box 1302
San Francisco, CA 94143-1302
Tel.: (415) 206-8854
Fax: (415) 206-6997
Email: matilda.chan@ucsf.edu

Table S1. Sequence of primers used for qPCR

Name of primer	Sequence 5' to 3'
CCR2 Forward	ACAGCTCAGGATTAACAGGGACTTG
CCR2 Reverse	ACCACTTGCATGCACACATGAC
CCL2 Forward	TTAAAAACCTGGATCGGAACCAA
CCL2 Reverse	GCATTAGCTTCAGATTTACGGGT
VEGFA Forward	CCAGACCTCTCACCGGAAAG
VEGFA Reverse	CTGTCAACGGTGACGATGATG
VEGFB Forward	GAGATGTCCCTGGAAGAACACA
VEGFB Reverse	GAGTGGGATGGGTGATGTCAG
HPRT Forward	GCCTAAGATGAGCGCAAGTTG
HPRT Reverse	TACTAGGCAGATGGCCACAGG

Supplemental Figure 1: Figure 8 Full-size blots

A.

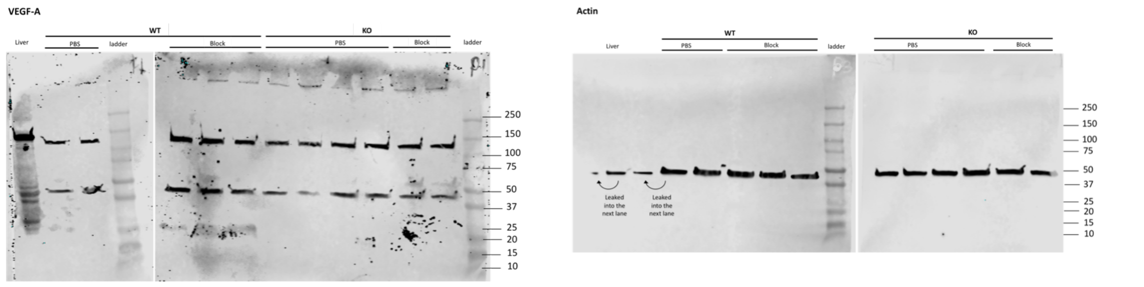

B.

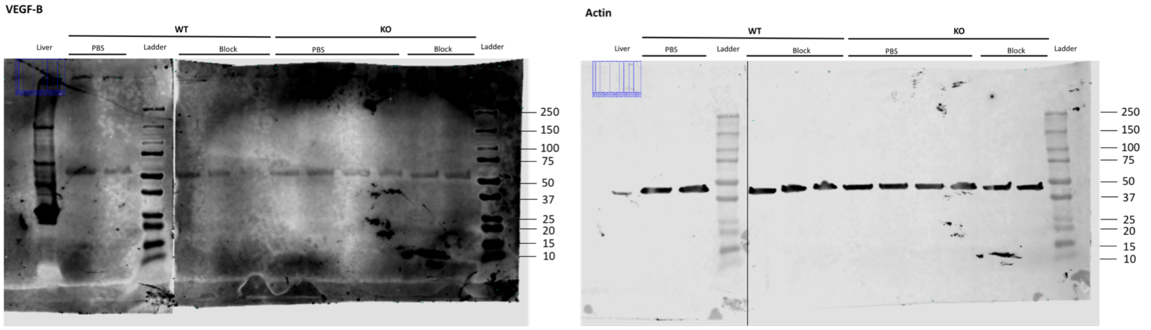

C.

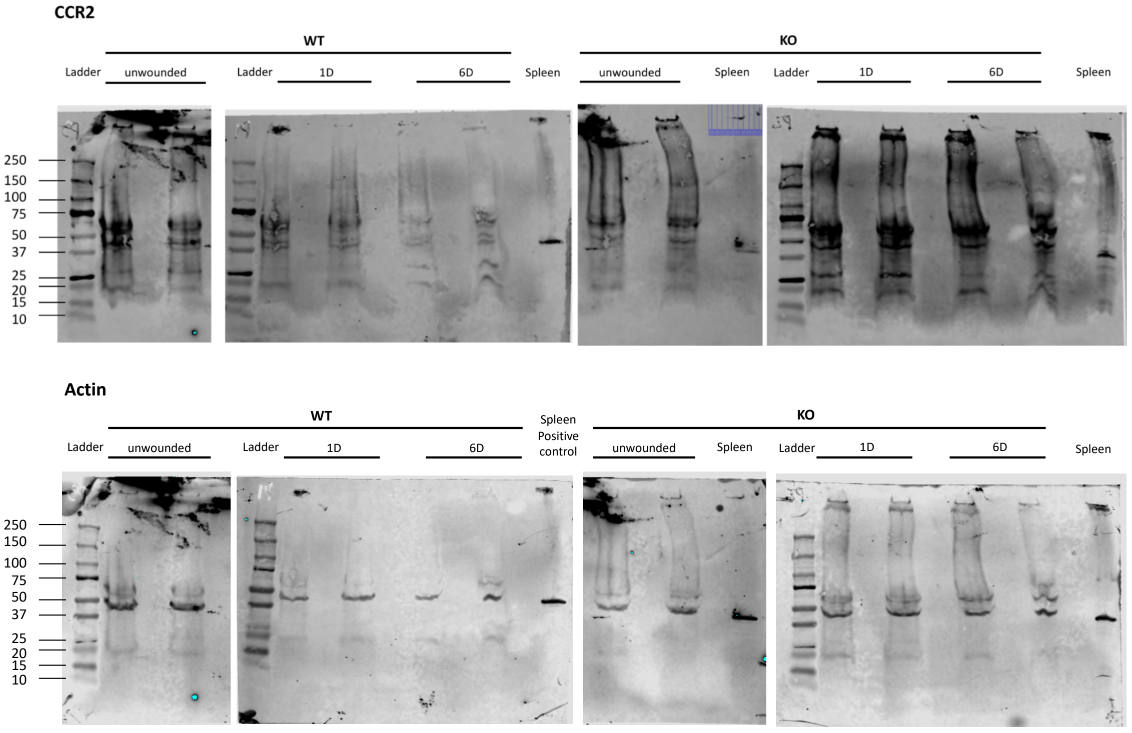

Supplement: Supplementary file 1 — Supplementary Information [file 41598_2019_47831_MOESM1_ESM.pdf]
